# The UK National Prolapse Survey: 10 years on

**DOI:** 10.1007/s00192-017-3476-3

**Published:** 2017-09-15

**Authors:** Swati Jha, Alfred Cutner, Paul Moran

**Affiliations:** 1 Urogynaecology Department, Jessop Wing, Sheffield Teaching Hospitals NHS Foundation Trust, Sheffield, S10 2SF UK; 2 Urogynaecology Unit, Elizabeth Garrett Anderson Hospital, University College London Hospitals, London, WC1E 6DH UK; 3 Department of Urogynaecology, Worcester Royal Hospital, Charles Hastings Way, Worcester, WR 5 1DD UK

**Keywords:** Pelvic organ prolapse, Cystocele, Rectocele, Vault prolapse, Vaginal mesh, Laparoscopic urogynaecology

## Abstract

**Introduction and hypothesis:**

To assess trends in the surgical management of pelvic organ prolapse (POP) amongst UK practitioners and changes in practice since a previous similar survey.

**Methods:**

An online questionnaire survey (Typeform Pro) was emailed to British Society of Urogynaecology (BSUG) members. They included urogynaecologists working in tertiary centres, gynaecologists with a designated special interest in urogynaecology and general gynaecologists. The questionnaire included case scenarios encompassing contentious issues in the surgical management of POP and was a revised version of the questionnaire used in the previous surveys. The revised questionnaire included additional questions relating to the use of vaginal mesh and laparoscopic urogynaecology procedures.

**Results:**

Of 516 BSUG members emailed, 212 provided completed responses.. For anterior vaginal wall prolapse the procedure of choice was anterior colporrhaphy (92% of respondents). For uterovaginal prolapse the procedure of choice was still vaginal hysterectomy and repair (75%). For posterior vaginal wall prolapse the procedure of choice was posterior colporrhaphy with midline fascial plication (97%). For vault prolapse the procedure of choice was sacrocolpopexy (54%) followed by vaginal wall repair and sacrospinous fixation (41%). The laparoscopic route was preferred for sacrocolpopexy (62% versus 38% for the open procedure). For primary prolapse, vaginal mesh was used by only 1% of respondents in the anterior compartment and by 3% in the posterior compartment.

**Conclusion:**

Basic trends in the use of native tissue prolapse surgery remain unchanged. There has been a significant decrease in the use of vaginal mesh for both primary and recurrent prolapse, with increasing use of laparoscopic procedures for prolapse.

**Electronic supplementary material:**

The online version of this article (10.1007/s00192-017-3476-3) contains supplementary material, which is available to authorized users.

## Introduction

The first UK prolapse survey was conducted in 2006. It highlighted widespread variations in practice in the surgical management of prolapse [1]. A second survey was conducted 5 years later [2] and demonstrated a consistent rise in the use of vaginal mesh with little change in the use of other surgical procedures for pelvic organ prolapse (POP). The risk of recurrent prolapse and the need for further surgery were responsible for the increase in mesh usage [3–5]. Following US Food and Drug Administration (FDA) approval for vaginal mesh for POP in 2002, there was a significant increase in mesh procedures for both primary and recurrent prolapse, and recurrence rates were quoted as the reason for this increase. Recent studies suggest that the risk of recurrent prolapse in the same compartment after native tissue repair has been overestimated and is more likely to be in the region of 10% [6–9].

The aim of repeating the National Prolapse Survey was to assess trends in the surgical management of POP amongst UK practitioners and changes in practice since the first survey. The use of vaginal mesh in light of the recent publications on mesh usage and the use of laparoscopic urogynaecology procedures for POP were also assessed. We anticipated that the use of vaginal mesh for both primary and recurrent prolapse would have declined.

## Materials and methods

This was an online electronic questionnaire survey (Typeform Pro). The questionnaire was developed and trialled by each author to ensure that it worked smoothly and allowed selection of different responses. The first questionnaire was made available online in December 2016 and three reminders were emailed at intervals of 5 weeks with the last one in April 2017. All duplicate responses were removed from the analysis.

The questionnaire followed the same structure as that of the initial survey in 2006 which was developed following a pilot study that included the seven consultant gynaecologists at Worcester Royal Hospital, UK. The method of questionnaire development is given in the initial article that provides details of the survey [1]. Case scenarios formulated for the first survey were modified to incorporate a further range of options taking into account current practice trends in surgical correction of prolapse. The final questionnaire included questions on the management of anterior vaginal wall prolapse (AWP, question 1), uterine prolapse in conjunction with vaginal wall prolapse (UVP, question 2), posterior vaginal wall prolapse (PWP, question 3), and vaginal vault prolapse (VVP, question 4), as in the previous survey. A section regarding the use of both abdominal and vaginal mesh was added. Respondents were also asked how they classified POP and the degree of follow-up patients received following prolapse surgery, and if they were using the British Society of Urogynaecology (BSUG) database for auditing the results of their surgery. The survey is presented here as Appendix 1.

The online link to the questionnaire was emailed to all BSUG members. This ensured that all gynaecologists contacted had a urogynaecology practice. A covering email describing the objectives of the study accompanied the link to the questionnaire. The link to the questionnaire was emailed a further three times at intervals of 5 weeks.

Formal ethical approval was not required for the study as the survey was a review of clinicians’ practice.

The overall rates of response to each individual question were compared with the results of the previoius surveys in 2011 and 2006 using the chi-squared test and the significance of differences in the response rates was determined (*p* values <0.05 were considered statistically significant). The responses of groups A (urogynaecologists) and group B (gynaecologist with a special interest in urogynaecology) were compared and the responses of groups A and group C (generalists) were also compared, as in the previous surveys. Urogynaecologists (group A) included subspecialty-trained urogynaecologists or those whose workload included over 50% of urogynaecology. Special interest urogynaecologists (group B) included urogynaecologists with a significant but less than 50% workload in urogynaecology. Generalists (group C) included gynaecologists undertaking prolapse work.

## Results

Of 516 BSUG members contacted, 301 accessed the questionnaire and 212 returned a completed questionnaire for analysis giving a usable response rate of 41%. The rate of usable responses was better than in the 2011 and 2006 surveys in which the response rates were 35% and 28%, respectively. Of the completed questionnaires received, 32% (68/212) were from urogynaecologists (group A), 59% (125/212) were from gynaecologists with a special interest in urogynaecology (group B) and 5% (11/212) were from general gynaecologists (group C); 4% (8/212) did not specify the respondent’s designation. The questionnaires in which the respondent’s designation was not specified were included in the overall analysis, but were excluded from the analysis comparing the three target groups.

### Anterior wall prolapse

For AWP, 92% of respondents performed anterior colporrhaphy as the procedure of choice. This was a significant change (*p* < 0.0001) from 2011 when 71% performed anterior colporrhaphy as the procedure of choice. In women with concomitant urodynamic stress incontinence, 63% of respondents would perform continence surgery concurrently. Only 1% used a graft for primary prolapse, compared with 11% in 2011, whereas 16% would do so for a recurrent AWP either alone or in combination with fascial plication compared with 56% in 2011. The proportions of respondents using synthetic and biological mesh usage was similar to those found in 2011. In women with proven urodynamic stress incontinence, 63% of respondents would perform a concurrent continence procedure at the time of the prolapse repair, whereas 37% would not do so concurrently. These results are shown in Table 1.

**Table 1:** Question 1: Anterior vaginal wall prolapse

		2006	2011	Current
Procedure of choice for primary repair	Anterior colporrhaphy	77%	71%	92%
	Graft ± fascial plication	10%	11%	1%
		24% synthetic	52% synthetic	1 respondent synthetic
		76% biological	48% biological	1 respondent biological
	Paravaginal repair	6%	9%	3%
	Other	7%	9%	4%
Would a continence procedure be performed concurrently	Yes	–	–	63%
	No	–	–	37%
Procedure of choice for recurrence	Anterior colporrhaphy	45%	21%	65%
	Graft ± fascial plication	34%	56%	16%
		28% synthetic	55% synthetic	52% synthetic
		72% biological	45% biological	48% biological
	Paravaginal repair	15%	11%	11%
	Other	6%	12%	4%
Would surgery be undertaken in women whose family is incomplete	Yes	44%	48%	31%
	No	56%	52%	69%

Comparing groups A, B and C, 92%, 90% and 63% of respondents, respectively, performed anterior colporrhaphy. Group A and B respondents were therefore significantly more likely to perform anterior repair than group C respondents (92% vs. 63%, *p* = 0.01; 90% vs. 63%, *p* < 0.001). The numbers of respondents in the three groups using mesh for primary prolapse were so low that the use of mesh could not be analysed. For secondary redo anterior wall repairs, 13%, 15% and 0% of group A, B and C respondents, respectively, would use a graft either alone or in combination with fascial plication. The difference in the use of graft for secondary repairs was not significantly different between groups A and B. The proportions of respondents in all three groups who would perform a concurrent continence procedure were similar (group A 60%, group B 61%, group C 72%). The proportions of respondents in all three groups who would operate on women who had not completed their family were also similar (group A 32%, group B 27%, group C 45%; not significantly different).

Compared with the two previous surveys, the greatest change in practice was the significant reduction in mesh usage for primary and recurrent repairs (*p* < 0.002). There also seems to have been a revival in the use of anterior vaginal wall repair using native tissue as the procedure of choice for both primary and recurrent anterior compartment prolapse.

### Uterovaginal prolapse

The second question assessed trends in the surgical management of second degree uterine prolapse in conjunction with AWP. In women with UVP, 75% of respondents would still perform vaginal hysterectomy combined with vaginal wall repair as the procedure of choice, but this was not a significant change from the previous surveys. The proportion of respondents who would perform a sacrohysteropexy (SHP) was 10%. The method of choice for vault support during a vaginal hysterectomy had shifted from attaching the uterosacral ligaments to the vault to a McCall culdoplasty. In the current survey, 37% of respondents would operate in women whose family was incomplete compared with 35% in 2011 and 26% in 2006, and the procedure of choice was still SHP. These results are shown in Table 2.

**Table 2:** Question 2: Uterovaginal wall prolapse (stage II)

		2006	2011	Current
Preoperative urodynamics	Yes	70%	59%	9%
	No	30%	41%	91%
Procedure of choice	Vaginal hysterectomy + repair	82%	82%	75%
	Other	18%	18%	25%^a^
Method of vault support intraoperatively	Suturing uterosacral ligaments to the vault	63%	56%	39%
	McCall culdoplasty	13%	16%	33%
	Sacrospinous fixation	19%	20%	22%
	Other	5%	8%	6%
Management of women whose family is incomplete	Ring pessary	68%	58%	Operate: yes 37%, no = 63%
	Advise against pregnancy and Vaginal hysterectomy + repair	2%	<1%	
	Uterus preservation surgery	24%	34%	
	Refer	6%	7%	

^a^Sacrohysteropexy 10%, sacrospinous hysteropexy 8%, subtotal hysterectomy and sacrocervicopexy 4%, Manchester repair 2%, other 1%

Of all respondents, 55% would perform SHP, and of those performing the procedure, 62% would offer it routinely. All respondents offering SHP as a primary procedure for prolapse would perform the procedure laparoscopically. For 67% of respondents the method of choice was the wrap-around technique through the broad ligament and anchored to the cervix anteriorly, and for the remaining 33% the method of choice anchoring the mesh posteriorly to the cervix and upper vagina.

Comparing surgical practice in the management of UVP, similar proportions of respondents in the three groups would perform preoperative urodynamics (group A 13%, group B 36%, group C 9%). Similar proportions of respondents would perform vaginal hysterectomy and repair as the procedure of choice (group A 73%, group B 76%, group C 46%). Similar proportions of respondents would also perform McCall culdoplasty as the procedure of choice for supporting the vault (group A 31%, group B 24%, group C 18%). Uterus-preserving surgery was the procedure of choice in 16%, 18% and 36% of group A, B and C respondents, respectively, and SHP was the procedure of choice in 15%, 5% and 36% of respondents, respectively.

Comparing the results with those of the previous surveys, there was a significant increase in the proportion of respondents who would perform SHP as the procedure of choice from only 1% in the previous surveys (*P* = 0.005). Vaginal hysterectomy and repair was still the most commonly performed procedure with no change in the proportion of respondents. In the current survey, only 9% of respondents would undertake urodynamic investigations prior to prolapse surgery even in the presence of stress urinary incontinence, in comparison with 59% in 2011 and 70% in 2006.

### Posterior vaginal wall prolapse

The third question assessed the surgical trends in the management of PWP. In women with PWP, 97% of respondents would perform posterior colporrhaphy with midline fascial plication as the procedure of choice for primary prolapse and 62% for recurrent prolapse. For primary and recurrent PWP, 3% and 11% of respondents, respectively, would use a graft. In the current survey, 51% of respondents would change the procedure if the patient was sexually active, in comparison with 14% in 2011 and 11% in 2006. These results are shown in Table 3.

**Table 3:** Question 3: Posterior vaginal wall prolapse

		2006	2011	Current
Procedure of choice	Posterior colporrhaphy	75%	66%	97%
	Graft ± fascial plication	9%	12%	3%
		40% synthetic	45% synthetic	29% synthetic
		60% biological	55% biological	71% biological
	Site-specific repair	11%	18%	–
	Other	5%	4%	–
Would colorectal opinion be sought or anorectal studies performed in the presence of defaecatory symptoms	Yes	15%	16%	24%
	No	85%	84%	76%
Procedure of choice for recurrence	Posterior colporrhaphy	38%	23%	63%
	Mesh ± fascial plication	49%	49%	11%
		44% synthetic	53% synthetic	41% synthetic
		56% biological	47% biological	59% biological
	Site-specific repair	6%	14%	26%
	Other	7%	14%	
Would the procedure change if the patient is not sexually active	Yes	11%	14%	51%
	No	89%	86%	49%

The proportions of respondents who would refer for anorectal studies was not significantly different among the three groups (group A 29%, group B 22%, group C 0%). Similar proportions of respondents would perform posterior colporrhaphy with midline fascial plication as the procedure of choice (group A 95%, group B 99%, group C 63%). For primary repairs, 3%, 0% and 36% of group A, B and C respondents, respectively, would use a graft alone or in combination with fascial plication. Significantly more group C respondents would use a graft for primary repair compared with group A and B respondents (36% vs. 3% and 0%, *p* < 0.001). However, there was no difference in graft usage between groups A and B. For recurrent PWP, the procedure of choice was a native tissue repair (group A 63%, group B 63%, group C 36%). A graft-reinforced repair for recurrent prolapse was performed by 11%, 8% and 36% group A, B and C, respondents, respectively.

Compared with the previous surveys, there was a significant reduction in the use of mesh for both primary and recurrent PWP and a greater use of native tissue repair in both groups. Respondents seemed to be more cautious when operating on women who are sexually active. Rates of referral to a colorectal surgeon for defaecatory problems remained constant.

### Vaginal vault prolapse

The fourth question assessed the management of VVP. In the current survey, 82% of the respondents would operate on a VVP. For primary VVP, 54% of respondents would perform sacrocolpopexy (SCP), and 41% a repair and sacrospinous fixation (SSF) as the procedure of choice. In women with previous vaginal hysterectomy for prolapse or a recurrent VVP, 75% of respondents would perform SCP and 23% SSF as the procedure of choice. The procedure of choice was more likely to change if the patient was sexually active. These results are shown in Table 4.

**Table 4:** Question 4: Vault prolapse

		2006	2011	Current
Refer or operate	Operate	66%	86%	82%
	Refer	34%	14%	18%
Procedure of choice	Anterior + posterior repair	28%	20%	2%
	Abdominal sacrocolpopexy	38%	44%	54%
	Sacrospinous fixation ± repair	19%	26%	41%
	Prespinous fixation ± repair	1%	–	–
	Posterior intravaginal slingplasty ± repair	6%	10%	3%
	Uterosacral ligament fixation + repair	3%	–	–
	Other	5%	–	–
Would procedure of choice change if patient not sexually active^a^	Yes	16%	11%	51%
	No	84%	89%	49%

^a^Expressed as percentages of those who would operate

Of those respondents who would perform SCP, 62% would do so laparoscopically and the remaining 38% as an open procedure. The proportions of respondents in the three groups who would operate varied (group A 88%. group B 82%, group C 63%; group A vs. group C, *p* < 0.001; group A vs. group B, *p* = 0.3, not significant). For primary VVP, 66% and 55% of group A and C respondents, respectively, would perform SCP as the procedure of choice. Of group B respondents, 44% would perform vaginal wall repair and 50% SSF as the procedure of choice. For recurrent VVP, 85%, 68% and 64% of group A, B and C respondents, respectively, would perform SCP as the procedure of choice. Of those performing SCP, the laparoscopic route was preferred by 66%, 51% and 100% of group A, B and C respondents, respectively. The patient’s sexual status would influence the procedure chosen by 44%, 57% and 36% of group A, B and C respondents, respectively, but these rates were not significantly different among the three groups.

Compared with previous surveys, there was no significant change in the proportions of respondents who would perform SCP or SSF for primary VVP. However, there was a significant reduction in the proportion of repondents who would undertake just an anterior and posterior vaginal wall repair and a significant increase in the proportion who would change their choice of procedure based on the patient’s sexual activity.

### Mesh users

Of all respondents, 75% used mesh abdominally or vaginally, and only 5% used vaginal mesh for primary prolapse in any compartment. The numbers of mesh procedures performed by individual respondents was variable, with the majority doing fewer than 20 procedures annually. All the different mesh procedures performed and numbers of each performed annually are shown in Table 5.

**Table 5:** Mesh procedures and numbers performed annually

Procedure	No. (%) of respondents performing the procedure	Number of procedures performed^a^		
		<5	5–20	>20
Open sacrocolpopexy	106 (50)	47%	40%	13%
Laparoscopic sacrocolpopexy	74 (35)	29%	54%	17%
Open sacrohysteropexy	62 (30)	68%	27%	5%
Laparoscopic sacrohysteropexy	63 (30)	38%	53%	15%
Total laparoscopic hysterectomy + sacrocolpopexy	14 (6)	44%	44%	22%
Subtotal hysterectomy + sacrocervicopexy	46 (22)	57%	37%	6%
Vaginal mesh (any compartment)	43 (20)	41%	47%	12%

^a^Expressed as percentages of those performing the procedure

The method of classification used for prolapse varied. In all three groups, most respondents used the Pelvic Organ Prolapse Quantification (POP-Q) system [10](67%, 55% and 63% of group A, B and C respondents, respectively). Of all respondents, 92% saw their patients in the gynaecology outpatient clinic at 6 weeks to 6 months after surgery, and 74% used the BSUG database to audit the results of their surgery.

## Discussion

The past 5 years have seen a significant decline in the use of vaginal mesh for both primary and recurrent prolapse with an increase in the use of native tissue repair as the procedure of choice for both the anterior and the posterior compartment. Vaginal hysterectomy and repair continues to be the most commonly performed procedure for uterine prolapse in conjunction with a cystocele. There has been an increase in the number of uterus-conserving operations performed for uterine prolapse with 10% of respondents performing SHP as the procedure of choice for uterine prolapse. There has also been a significant increase in laparoscopic urogynaecology procedures with almost all SHP and a majority of the SCP being performed laparoscopically.

The usable response rate (41%) was better than in the previous national surveys. This could be because a more focused group of gynaecologists performing prolapse surgery were approached and fewer questionnaires were sent out in this survey than in the previous ones. As prolapse surgery is becoming more specialized there may have been fewer generalists responding compared with the previous surveys. Although the total membership of the BSUG is 516, many of these members are retired or are associate or trainee members; hence the response rate is reflective of current practice.

In this survey specialized surgery such as SHP and SCP was performed more frequently by generalists than in the previous survey. This is probably because generalists undertaking this work are BSUG members rather than because all generalists are undertaking this specialized surgery which includes both uterus-conserving surgery and laparoscopic urogynaecology procedures.

Several recent reports have led to a huge decline in the use of vaginal mesh and this explains why this survey found almost complete cessation of vaginal mesh use for primary prolapse and a significant reduction in its use for recurrent prolapse. In 2012, the Medicines and Healthcare Products Regulatory Agency funded ‘Summaries of the Safety/Adverse Effects of Vaginal Tapes/Slings/Meshes for Stress Urinary Incontinence and Prolapse’ otherwise known as the ‘York Report’ [11, 12]. This identified pain, dyspareunia, deterioration in sexual function, organ damage and mesh exposure and erosion as some of the serious complications associated with the use of mesh. In a meta-analysis published in 2016 [13], 37 randomized controlled trials comparing women who underwent transvaginal graft repair (*n* = 1,986) and traditional native tissue repair (*n* = 2,037) were reviewed. Compared with women who underwent native tissue repair, women who underwent synthetic non-absorbable mesh repair were less likely to be aware of prolapse at 1 to 3 years (RR 0.66, 95% CI 0.54 to 0.81), less likely to have recurrent prolapse on examination (RR 0.40, 95% CI 0.30 to 0.53) and less likely to require repeat surgery for prolapse (RR 0.53, 95% CI 0.31 to 0.88). However, more women in the mesh group required repeat surgery for the combined outcome of prolapse, stress incontinence, or mesh exposure (RR 2.40, 95% CI 1.51 to 3.81). A Scottish population-based cohort study (1997–2016) reported 5-year outcomes in 18,986 women who underwent primary anterior or posterior repair, with mesh used in 7%. The study concluded that mesh procedures for anterior and posterior compartment prolapse cannot be recommended for primary prolapse repair [14].

Overall practice in the surgical management of POP amongst urogynaecologists and gynaecologists with a special interest in urogynaecology were similar yet varied in some respects from the practice of general gynaecologists. The degree of variation was less than that seen in the previous surveys probably because the generalists who completed the questionnaire were BSUG members and therefore had a practice similar to that of the other two groups.

In a 2015 survey of members of the International Urogynaecology Association following the FDA’s safety announcement, 45% of respondents reported decreased use of mesh, and 7% used transvaginal mesh for primary repair and 58% for recurrence [15]. This is greater than the usage of mesh for primary and recurrent prolapse found in our survey, but this may be because the IUGA survey was performed 2 years before the current survey and if repeated the results would likely demonstrate further reductions in vaginal mesh usage. The response rate in the IUGA survey was only 13% compared with 41% for the UK survey. In the IUGA survey, the preferred procedures for prolapse of the anterior and posterior compartments were anterior and posterior colporrhaphy, respectively, similar to the results of the UK survey. Vaginal hysterectomy and repair was the preferred procedure for uterine prolapse in both surveys but was more common in the IUGA survey than in the UK survey (93% versus 75%). For VVP, the preferred route of surgery in the IUGA survey was vaginal, but the preferred route in the UK survey was abdominal, with SCP being the procedure of choice.

The practice of performing urodynamics prior to routine prolapse surgery seems to have declined significantly since the previous surveys. We believe that this is due to greater caution with the use of midurethral slings as a result of the adverse publicity and to the overall reduction in concurrent prolapse and continence surgery as a result of increasing litigation. Both these factors negate the utility of preoperative urodynamics.

This study had several limitations. The questionnaire used was not validated. However, using a questionnaire that was similar in content to the previous two national prolapse surveys allowed comparisons and assessment of changes in practice with time. In order to remove the selection bias from the previous surveys which was sent to all gynaecologists in a database, we approached BSUG members who were likely to include clinicians with a significant urogynaecology practice. However, the BSUG membership is unlikely to be representative of clinical practice of surgeons who are generalists but undertake POP surgery. The generalists included in this survey are not representative of all general gynaecologists undertaking prolapse work. Another limitation was that clinicians were asked to estimate the number of procedures done rather than using a database to provide these data. They were also asked to state whether they were urogynaecologists, special interest urogynaecologists or generalists. Both of these limitations are potential sources of bias. It is usually recommended that for online questionnaire surveys, measures should be taken to negate the influence of nonresponse bias by either performing a sensitivity analysis of nonrespondents or conducting a multiple imputation analysis. However, this was felt to be impractical and would not have added value to the findings of the survey.

Following the rise and fall of the use of vaginal mesh in practice, its use now appears to have stabilized and current levels are reflective of adherence to National Institute for Health and Care Excellence guidance and recently published literature on mesh usage. There is likely to be further decline with time and given that most clinicians are doing only a few cases per year, there may be a concern relating to the maintainance of skills in these procedures. With the reduction in the use of vaginal mesh procedures, there has been a corresponding increase in the use of laparoscopic urogynaecology procedures.

The need and demand for prolapse surgery is increasing. The incidence of POP surgery ranges from 1.5 to 1.8 per 1,000 women years with a peak in women aged 60–69 years [16]. There continues to be a shift in the way we manage POP, and although the recommendations for management are now more evidence-based, better trials comparing the different methods of repair for individual compartments are still needed to address the dilemmas faced in the management of recurrence.

## Electronic supplementary material

ESM 1 (DOCX 53 kb)
