# Supplementary material for: The UK National Prolapse Survey: 10 years on
Source: Int Urogynecol J. 2017 Sep 15;29(6):795–801. doi: 10.1007/s00192-017-3476-3 (PMC5948287; doi:10.1007/s00192-017-3476-3)
Supplement: Supplementary file 1 — (DOCX 53 kb) [file 192_2017_3476_MOESM1_ESM.docx]

**Appendix 1.**

**UK National Prolapse Survey: 10 years on**

# Given are four scenarios encountered in our daily practice. Please state how you would manage these cases from the options given. There are no right or wrong answers.

**Case Scenario 1 (Anterior Compartment)**

**A 45 year old sexually active woman presents with an anterior wall prolapse to the introitus (Aa 0, Ba 0) but minimal uterocervical descent (C-4/D-6). She is fit and well, her family is complete and she has not had previous surgery.**

1. What would be your preferred surgical procedure in this scenario?
2. Anterior Colporrhaphy (midline Plication)
3. Paravaginal repair
4. Vaginal route
5. Abdominal route
6. Laparoscopic route
7. Anterior Mesh with no plication
8. Biological mesh inlay
9. Synthetic mesh inlay
10. Synthetic mesh kit

Which one? _____________________

1. Combination plication and graft inlay
2. Biological free mesh
3. Synthetic free mesh
4. Synthetic mesh kit

Which one? _____________________

1. Vaginal hysterectomy with repair
2. Abdominal Hysteropexy with or without repair
3. Laparoscopic Hysteropexy with or without repair
4. If the patient also had daily symptomatic stress incontinence and proven Urodynamic Stress Incontinence (with no detrusor overactivity and normal voiding studies) would you do an incontinence procedure at the same time?

Yes No

1. If the patient presents 10 years after her initial repair with recurrent prolapse, the same symptoms and findings (recurrent grade 2 cystocele with central and lateral defects and no incontinence)

Would you

1. Operate
2. Refer to a designated urogynaecologist or tertiary centre

If you operate which of the above procedures would you do from those above:

1

2 i, 2 ii 2iii

3 i 3ii 3iii

4 i 4 ii 4iii

5

6

7

1. If the patient was 30 and had not completed her family would you operate if physiotherapy had failed

Yes No

**Case Scenario 2 (Uterine prolapse)**

**A 65 year old fit and well para 2 woman presents with cystocoele (Aa 0; Ba +1) and uterovaginal prolapse to the introitus (C 0; D -3) with no significant rectocele. She has normal cervical cytology and a normal sized uterus with no incontinence or voiding problems.**

1. Would you perform preoperative urodynamics?

Yes No

1. What would be your procedure of choice
2. Vaginal hysterectomy and repair

If yes what vault support do you provide

1. Uterosacral plication
2. Ultra-High uterosacral incorporation
3. McCalls Culdoplasty
4. Sacrospinous fixation
5. Manchester repair
6. Sacrospinous Hysteropexy
7. Subtotal hysterectomy and sacrocervicopexy

Open Laparoscopic

1. Abdominal hysterectomy and Sacrocolpopexy

Open Laparoscopic

1. Sacrohysteropexy

Open Laparoscopic

1. Laparoscopic Hysterectomy with Uterosacral plication
2. Mesh reinforcement
3. Biological mesh inlay
4. Synthetic mesh inlay
5. Synthetic mesh kit

Which one? _____________________

1. If the patient was 35 had not completed her family and failed physiotherapy would you operate?

Yes No

If yes which of the above procedure would you perform?

2/ 3/ 6/ 7/ 8

1. If you perform Sacrohysteropexy
2. Do you offer this as a routine option?

Yes No

1. Do you offer hysteropexy as one of the choices for the following patients:
2. Family not complete

Yes No

1. Family complete but pre-menopausal

Yes No

1. Post menopausal

Yes No

1. Which Mesh do you use? _________________
2. Do you place your mesh:
3. On the posterior cervix/upper vaginal
4. Mesh rap through the broad ligament and anchored to the cervix anteriorly

**Case Scenario 3 (Posterior Compartment)**

**A 48 year old sexually active woman has a main complaint of a rectocele reaching the introitus (Ap 0; Bp 0). She describes a need to sometimes use vaginal splinting to evacuate her bowel. She has no other anorectal symptoms. There is no uterine or anterior vaginal prolapse. She is fit and well, her family is complete and she has not had previous surgery.**

1. Would you refer for a colorectal opinion/ano-rectal studies prior to offering her surgery?

Yes No

b) Your preferred surgical procedure here would be-

1. Posterior Colporrhaphy (midline Plication)
2. Posterior Mesh with no plication
3. Biological mesh inlay
4. Synthetic mesh inlay
5. Synthetic mesh kit

Which one? _____________________

1. Combination plication and graft inlay
2. Biological mesh inlay
3. Synthetic mesh inlay
4. Synthetic mesh kit

Which one? _____________________

c) If the patient presents 10 years after her initial repair with recurrent prolapse, the same symptoms and findings (recurrent grade 2 rectocele)

Would you

1. Operate
2. Refer to a designated urogynaecologist or tertiary centre

If you operate which of the above procedures would you do from those above:

1

2 i

ii

iii

3 i

ii

iii

d) Would your technique change if the patient was sexually active?

Yes No

**Case Scenario 4 (Vault Prolapse)**

**A 56 year old presents with a post hysterectomy primary vault prolapse reaching the introitus (Ba, C, and Bp all 0). She had a TAH at 45 for menorrhagia. She is fit and well, has no urinary incontinence.**

1. Would you
2. Operate
3. Refer to a designated urogynaecologist or tertiary centre

**If you would refer move straight to the final section.**

1. Your preferred surgical procedure here would be
2. Anterior + Posterior repair/perinorrhaphy
3. Vaginal wall repair + Sacrospinous fixation
4. Vaginal Wall repair + Bilateral Illiococcygeal fixation
5. Vaginal Mesh
6. Biological Graft inlay
7. Synthetic type inlay
8. Synthetic Mesh kit

Which one? _____________________

1. Sacrocolpopexy

Open Laparoscopic

Which Mesh do you Use for Sacrocolpopexy? ________________________

1. If the same patient had previous vaginal hysterectomy and repair for prolapse and this was a recurrent prolapse of the vault what would be your preferred surgical procedure from the list above?

1

2

3

4 i

ii

iii

Which one?

5

Open Laparoscopic

**Mesh users**

How many of each have you done in the past year (12 months) ?

**Abdominal Sacrocolopexy : Open**

**Laparoscopic**

**Sacrohysteropexy: Open**

**Laparoscopic**

**Total hysterectomy and sacrocolpopexy as a treatment for uterine prolapse**

**Subtotal hysterectomy and sacrocervicopexy as a treatment for uterine prolapse**

**Vaginal Mesh**

Anterior mesh (biological free graft)

Anterior mesh (synthetic free graft)

Anterior mesh Kit (synthetic mesh kit)

Posterior mesh (biological free graft)

Posterior mesh (synthetic free graft)

Posterior mesh Kit (synthetic mesh kit)

Vault mesh (biological free graft)

Vault mesh (synthetic free graft)

Vault mesh Kit (synthetic mesh kit)

**Do you use vaginal mesh insertion for primary vaginal prolapse?**

**Y N**

# Final Section

# 1. Would you class yourself as :

Generalist / Gynaecologist with a designated special interest in urogynaecology / Urogynaecologist

# 2. How do you classify ‘prolapse’ in your routine NHS practice?

Small/medium/large

Grade 1, 2 and 3

First, second, third and fourth degree (procidentia or complete eversion)

Baden/Walker six point assessment

POP-Q scoring/measurement

Other:…………………………..

3. Do you see patients for follow up who have undergone prolapse surgery?

Yes No

YES: When postoperatively 6wks / 3 months / 6 months

4. Do you use the BSUG database to audit the results of your surgery. Yes No
